# Comparative Genomic Analysis of a Novel Strain of Taiwan Hot-Spring Cyanobacterium *Thermosynechococcus* sp. CL-1

**DOI:** 10.3389/fmicb.2020.00082

**Published:** 2020-01-31

**Authors:** Yen-I Cheng, Lin Chou, Yi-Fang Chiu, Hsin-Ta Hsueh, Chih-Horng Kuo, Hsiu-An Chu

**Affiliations:** ^1^Institute of Plant and Microbial Biology, Academia Sinica, Taipei, Taiwan; ^2^Sustainable Environment Research Laboratories, National Cheng Kung University, Tainan, Taiwan

**Keywords:** cyanobacterium, *Thermosynechococcus*, genome, comparative genomics, thermophilic cyanobacterium, adaption

## Abstract

*Thermosynechococcus* is a genus of thermophilic unicellular cyanobacteria that are dominant in microbial mats at about 50–65°C in alkaline hot springs of eastern Asia. We used PacBio SMRT Sequencing to sequence the complete genome of a novel strain of thermophilic cyanobacterium, *Thermosynechococcus* sp. CL-1, isolated from the Chin-Lun hot spring (pH 9.3, 62°C) in Taiwan. Genome-scale phylogenetic analysis and average nucleotide identity (ANI) results suggested that CL-1 is a new species in the genus *Thermosynechococcus*. Comparative genome analysis revealed divergent genome structures of *Thermosynechococcus* strains. In addition, the distinct genetic differences between CL-1 and the other *Thermosynechococcus* strains are related to photosynthesis, transporters, signal transduction, the chaperone/usher system, nitric oxide protection, antibiotic resistance, prokaryotic immunity systems, and other physiological processes. This study suggests that *Thermosynechococcus* strains have actively acquired many putative horizontally transferred genes from other bacteria that enabled them to adapt to different ecological niches and stressful conditions in hot springs.

## Introduction

Thermophilic cyanobacteria grow photosynthetically under high-temperature and very stressful environments in hot springs (Ward et al., 2012). The underlying mechanisms of how thermophilic cyanobacteria adapt to different stressful conditions of hot springs are still not fully understood. In addition, the enzymes of hot-spring cyanobacteria are highly stable and can catalyze enzymatic reactions under high-temperature conditions (Patel et al., 2019). Therefore, hot-spring cyanobacteria and their bioproducts may have high value for biotechnology and industrial applications (Patel et al., 2019).

Genomic studies of hot-spring cyanobacteria have been conducted in several countries such as the United States and Japan (Nakamura et al., 2002; Bhaya et al., 2007; Stolyar et al., 2014; Olsen et al., 2015). Molecular analysis of the microbial mat community in Octopus Spring of Yellowstone National Park revealed three unrelated *Synechococcus* phylogenetic lineages (>10% 16S rRNA sequence variation), A/B, C1, and C9 (Papke et al., 2003). The dominant *Synechococcus* in the Yellowstone hot springs were the *Synechococcus* A/B genotypes. Representative strains among A/B genotypes with sequenced genome information are JA-3-3-Ab and JA-2-3Ba (Bhaya et al., 2007). A recent comparative genomic study of four *Synechococcus* strains of Mushroom Spring, Yellowstone National Park, within the A lineage revealed distinct differences in gene content and alleles between high-light- and low-light-adapted strains (Olsen et al., 2015). This study suggested that strains of closely related putative ecotypes have developed different genomic adaptations that enable them to inhabit distinct ecological niches in microbial mats of Yellowstone hot springs.

*Thermosynechococcus* were dominant in microbial mats at about 50–65°C in alkaline hot springs of eastern Asia and also found at low abundance in some hot springs of North American (Papke et al., 2003; Liao et al., 2006; Everroad et al., 2012; Tang et al., 2018). Representative strains with sequenced genome information are *Thermosynechococcus elongatus* BP-1 (BP-1), *Thermosynechococcus vulcanus* NIES-2134 (*T. vulcanus*) and *Thermosynechococcus* sp. NK55 (NK55) isolated from hot springs in Japan (Nakamura et al., 2002; Stolyar et al., 2014); *T. elongatus* PKUAC-SCTE542 (SCTE542) isolated from a hot spring in western Sichuan in China (Liang et al., 2019); and *Synechococcus lividus* PCC6715 isolated from a hot spring in Yellowstone National Park in the United States (Dyer and Gafford, 1961). *Thermosynechococcus* strains have been widely used for photosynthesis and other scientific research (Liang et al., 2019; Patel et al., 2019). In addition, previous studies demonstrated that several *Thermosynechococcus* strains (BP-1, *T. vulcanus* and SCTE542) could perform natural transformation of foreign DNA via homologous recombination (Iwai et al., 2004; Liang et al., 2019).

**FIGURE 1:**
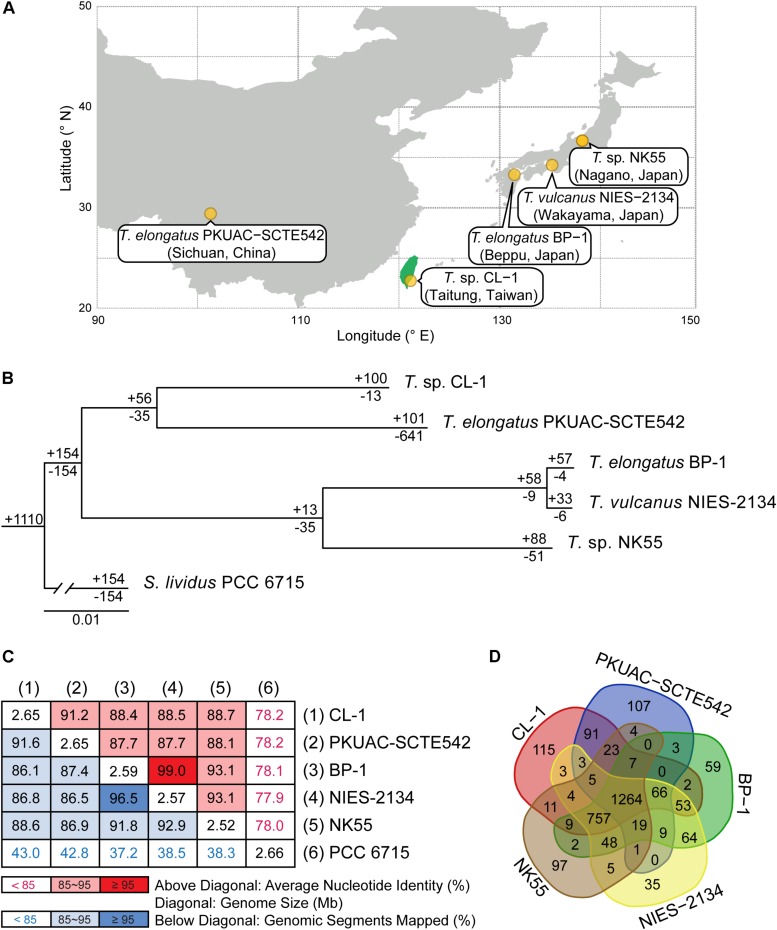
Comparisons between CL-1 and other representative *Thermosynechococcus* strains. **(A)** Geographic locations of isolation. **(B)** Maximum-likelihood phylogeny and phylogenetic distribution of homologous gene clusters. The molecular phylogeny was inferred by concatenated protein alignment of 1,085 single-copy genes shared by all genomes (281,438 aligned sites). All branches received 100% bootstrap support based on 1,000 replicates. *Synechococcus lividus* PCC 6715 was included as an outgroup to root the tree. The numbers above a branch and preceded by a “+” sign indicate the number of homologous genes uniquely present in all daughter lineages. The numbers below a branch and preceded by a “−” sign indicate the number of homologous genes uniquely absent in all daughter lineages. For example, the strain CL-1 has 100 homologous gene clusters that are not found in any of the other genomes and 13 gene clusters are shared by all genomes except CL-1. **(C)** Pairwise genome sequence similarity scores. The numbers along the diagonal indicate the genome sizes (Mb). The numbers above diagonal indicate the average nucleotide identity (ANI) values (%). The numbers below the diagonal indicate the proportion of segments that could be mapped between the two genomes for ANI calculation. **(D)** Number of shared and unique homologous gene clusters.

*Thermosynechococcus* sp. CL-1 (CL-1) was isolated in the Chin-Lun hot spring (pH 9.3, 62°C) in eastern Taiwan (Hsueh et al., 2007a; Figure 1A). The 16S rRNA gene of CL-1 was very similar (three to six mismatches) to that of representative *Thermosynechococcus* strains (BP1, *T. vulcanus*, NK55 and SCTE542). CL-1 has been studied in terms of CO_2_ elimination in the packed tower with potassium hydroxide to enhance mass transfer of CO_2_ (about fivefold) and also to regenerate alkaline solution by photosynthesis (Hsueh et al., 2007b). CL-1 showed high performance of carbon bio-fixation and also carbohydrate production (the highest content is 61%) under N-limiting conditions with sufficient dissolved inorganic carbon (Hsueh et al., 2009). In terms of types of bioreactors, CL-1 was applied to a continuous column cultivation system with 1.7 g/L/d of the highest cell mass productivity obtained (Su et al., 2012). The growth of CL-1 seems to be limited by illumination because its cell mass productivity can reach about 2.8 g/L/d under a 1.5-cm light path flat-plate photobioreactor and optimal biomass concentration (about 3 g/L) (Su et al., 2013). After modifying the composition ratios of medium, the cell mass productivity, CO_2_ fixation rate, and carbohydrate productivity can be up to 3.3, 5.3, and 1.8 g/L/d, respectively (Su et al., 2017). CL-1 seems to be a good candidate for CO_2_ elimination and simultaneous production of bioenergy precursor (such as carbohydrates) (Su et al., 2017).

Here we sequenced and analyzed the genome of CL-1 isolated from the Chin-Lun hot spring in Taiwan. The genome of CL-1 was compared to published genomes of four other *Thermosynechococcus* strains and *S. lividus* in the C1 genotype to reveal genetic diversity and adaption mechanisms of hot-spring cyanobacteria.

## Materials and Methods

### Sample Source and Genome Sequencing

The strain CL-1 was isolated from the Chin-Lun hot spring (pH 9.3, 62°C) in Taitung, Taiwan as described (Hsueh et al., 2007a). Axenic culture of the strain was performed at 45°C on a BG-11 agar plate or in BG-11 liquid medium supplemented with 20 mM TES (pH 8.0) under continuous white LED light (20 μmol photons m^–2^ s^–1^).

Total genomic DNA was extracted and purified by using the DNeasy Plant Maxi Kit (QIAGEN, Germany). Quality and quantity of purified genomic DNA were assessed by using the NanoDrop 2000 spectrophotometer (ThermoFisher, United States) and 1% agarose gel electrophoresis. Whole-genome shotgun sequencing involved using the PacBio Sequel platform (Pacific Biosciences, United States). One gel-plus (20 kb) library and one SMRT cell was used. The *de novo* genome assembly involved using the Hierarchical Genome Assembly Process (HGAP) assembler v4 (Chin et al., 2013). Gene prediction and annotation involved using the NCBI prokaryotic genome annotation pipeline (Tatusova et al., 2016). We also used the RAST annotation system to minimize poor calls (Aziz et al., 2008). All bioinformatics tools were used with the default settings unless stated otherwise.

### Genome Analysis

The procedures for genome analysis were based on those described in our previous work (Chung et al., 2013; Lo et al., 2013; Tsai et al., 2018). Briefly, the genome map was prepared by using Circos v0.69-6 (Krzywinski et al., 2009). To identify genes that may have originated from horizontal gene transfer (HGT), we performed BLASTP searches (Camacho et al., 2009) against the NCBI non-redundant protein database (Benson et al., 2018). Only hits with high-scoring pairs accounting for at least 90% of the query length and overall amino acid sequence similarity of at least 40% were retained to ensure that the hits represent likely homologs rather than non-homologous genes sharing only conserved domains. Genes with the best hit that lacked a taxonomic assignment at the genus level or was derived from metagenomics surveys were manually examined for more reliable inference. A gene is classified as putatively acquired if more than half of the top-five hits were from other genera. Among the putatively acquired genes, those with non-*Thermosynechococcus* sequences as the best hits are classified as being recently acquired.

For comparative analysis within the genus, a list of closely related strains with genome sequences available was compiled from the NCBI genome database (Benson et al., 2018) and a literature search (Table 1). The homologous gene clusters among these genomes were identified by using OrthoMCL (Li et al., 2003). The KEGG database (Kanehisa et al., 2010) was used for examining annotation and gene function. For pairwise genome alignments, the NUCleotide MUMmer (NUCmer) program of the MUMmer package v3.23 (Kurtz et al., 2004) was used with the setting “–maxmatch –mincluster 200.” The average nucleotide identity (ANI) and percentage of genome segments mapped for each genome pair were calculated by using FastANI (Jain et al., 2018). For phylogenetic analysis, MUSCLE v3.8.31 (Edgar, 2004) was used to generate multiple sequence alignments and PhyML v3.3 (Guindon and Gascuel, 2003) for maximum-likelihood inference. The packages ggplot2 v3.2.0 (Wickham, 2009) and gggenes v0.4.0 (Wilkins and Kurtz, 2019) were used to visualize gene locations and syntenies. The distributions of genes by functional category were plotted by using the “barplot” function of R (R Core Team, 2019).

**TABLE 1:** Genome statistics of representative thermophilic cyanobacteria.

Strain	*T.* sp.	*T. elongatus*	*T. elongatus*	*T. vulcanus*	*T.* sp.	*S. lividus*
	CL-1	PKUAC-SCTE542	BP-1	NIES-2134	NK55	PCC 6715
Origin	Taitung,	Sichuan,	Beppu,	Wakayama,	Nagano,	Yellowstone
	Taiwan	China	Japan	Japan	Japan	National Park,
						United States
GenBank accession	CP040671	CP032152	NC_004113	NZ_AP018202	NC_023033	NZ_CP018092
Genome size (bp)	2,647,823	2,648,728	2,593,857	2,571,271	2,520,064	2,659,739
G + C content (%)	53.5	53.3	53.9	53.9	53.8	53.5
Coding density (%)	88.9	48.9	89.5	88.7	85.9	76.6
Number of rRNA genes	3	3	3	3	3	3
Number of tRNA genes	41	41	42	40	41	41
Number of protein-coding genes	2,465	1,625	2,476	2,413	2,287	2,227
Number of pseudogenes	84	944	0	70	112	321

*All strains in the genus *Thermosynechococcus* with available genome sequences are included; *Synechococcus lividus* PCC 6715 was included as an outgroup. All strains were isolated from hot springs; the locations are listed.*

### Chemical Analysis

The water sample was taken from the Chin-Lun hot spring located in the east of Taiwan. Temperature, pH, and conductivity were *in situ* measured by probes. Other items, heavy metals, major ions, and non-purgeable organic carbon (NPOC) underwent laboratory analyses after standard pretreatments. The pretreatments and analyses were performed according to Taiwan National Institute of Environmental Analysis (NIEA) methods^1^. The analysis of As was performed by hydride generation/atomic absorption spectrometry. Mercury (Hg) was measured by cold-vapor atomic absorption spectrometry. Other heavy metals (Cr, Cd, Cu, and Zn) were measured by inductively coupled plasma-atomic emission spectrometry. Major ions (Na^+^, ${\text{NH}}_{4}^{+}$, K^+^, Mg^2+^, Ca^2+^, Cl^–^, ${\text{NO}}_{3}^{-}$, ${\text{PO}}_{4}^{3-}$, ${\text{HCO}}_{3}^{-}$/${\text{CO}}_{3}^{-}$, ${\text{SO}}_{4}^{2-}$) were measured by ion chromatography. NPOC was measured by the combustion oxidation/non-dispersive infrared absorption method.

## Results and Discussion

### Complete Genome Sequence of *Thermosynechococcus* sp. CL-1

**FIGURE 2:**
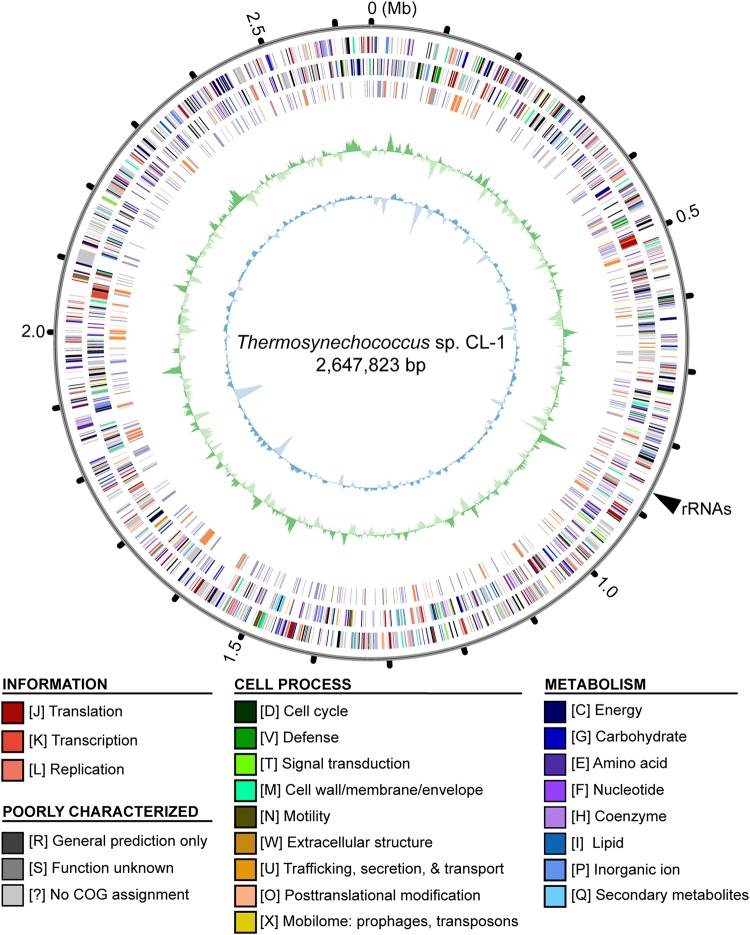
Genome map of *Thermosynechococcus* sp. CL-1. Rings from outside in: (1) Scale marks in Mb; (2, 3), protein-coding genes on the forward and reverse strand, respectively (color-coded by functional categories); (4) locations of putatively acquired genes (orange: recent; purple: other); (5) GC skew (positive: dark green; negative: dark green); (6) GC content (above average: dark blue; below average: light blue). The position of rRNA genes (at ∼0.87 Mb) is marked by a black triangle outside of the rings. Note that the regions with low GC content (e.g., at ∼0.14, 1.62, and 1.83 Mb) often correspond to putatively acquired genes in the “recent” category. These putatively acquired DNA segments may came from donor genomes with lower GC content, and have not been ameliorated yet due to their recent acquisition.

Whole-genome shotgun sequencing of *Thermosynechococcus* sp. CL-1 generated 47,666 filtered reads (average length = 7,536 bp, N50 length = 8,319 bp, total length = 359,209,730 bp). These reads provided ∼138-fold coverage of the genome, and the *de novo* assembly produced a single 2,647,823-bp circular chromosome (Figure 2). No plasmid was found. The annotation included 41 tRNA genes, one complete set of 16S-23S-5S rRNA genes, and 2,465 protein-coding genes (Table 1).

Similar to other cyanobacteria, such as the closely related *T. elongatus* BP-1 (Nakamura et al., 2002) or the more distant *Anabaena* sp. PCC 7120 (Kaneko et al., 2001), the chromosome organization did not exhibit strong patterns of GC-skew (Figure 2). One high positive peak at ∼0.87 Mb corresponded to the location of the rRNA gene cluster. Regarding the GC content, several low GC regions were found (e.g., at ∼0.14, 1.62, and 1.83 Mb). These regions all correspond to DNA segments that may have been acquired recently. These putatively acquired segments may have originated from donors with lower GC content and have not been ameliorated yet due to their recent acquisition.

### Comparison With Other Cyanobacteria

**FIGURE 3:**
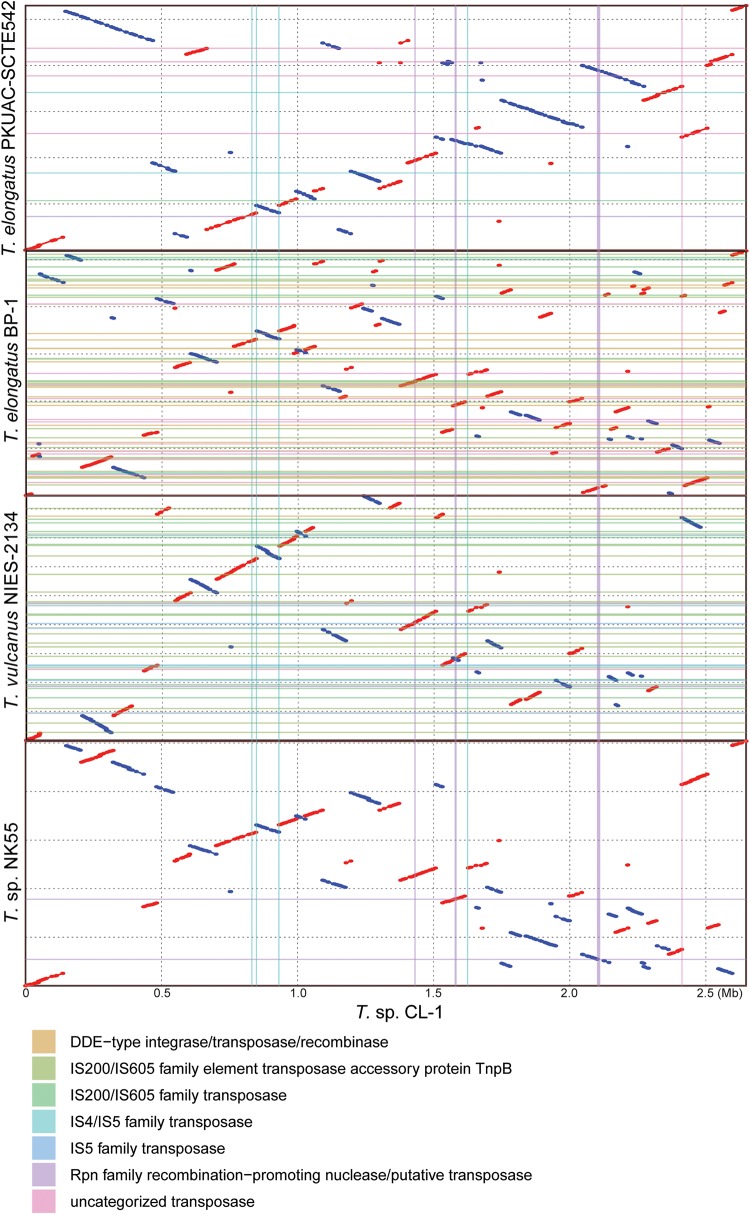
Pairwise genome alignments. The CL-1 genome is used as the reference for pairwise alignment with each of the other available *Thermosynechococcus* genomes. Red dots indicate matches in the same orientation, blue dots indicate matches in the opposite orientations. Mobilome-related segments (e.g., prophages, transposons, etc.) are represented by color lines (vertically for those found in the CL-1 genome and horizontally for those found in the other genomes) for visualization of correspondence between mobilome and chromosomal synteny breakpoints.

**FIGURE 4:**
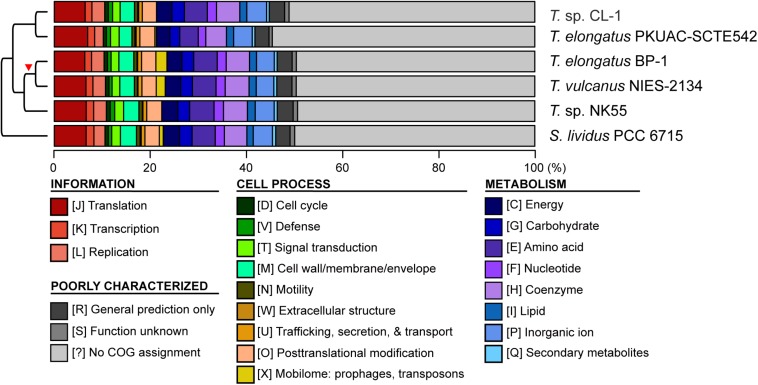
Functional classification of protein-coding genes. The functional categories were defined according to the COG database. The cladogram on the left side is based on Figure 1A. The red triangle indicates the putative origin of mobilome gene expansion.

Four other strains with complete genome sequences are available for the genus *Thermosynechococcus* (Table 1). We selected these genomes as well as the outgroup *S. lividus* for comparative analysis. Examination of gene content revealed 1,110 homologous gene clusters shared by all these genomes (Supplementary Table S1); 1,085 were present as single-copy genes in all strains. A concatenated alignment of these genes showed 281,438 aligned amino acid sites and produced a maximum-likelihood phylogeny with 100% bootstrap support for all branches (Figure 1B). From the genome-scale phylogeny, CL-1 from Taiwan is most closely related to SCTE542 from China, whereas the three other *Thermosynechococcus* strains from Japan form a sister clade. Intriguingly, two strains of *T. elongatus* (i.e., BP-1 and SCTE542) do not form a monophyletic clade, which indicates a conflict between phylogeny and taxonomy. Results from genome-wide ANI (Figure 1C) and the number of shared genes (Figure 1D) are similar to the patterns observed from molecular phylogeny. Pairwise genome alignments (Figure 3) indicated very low conservation in chromosomal organization among these cyanobacteria. BP-1 and *T. vulcanus* showed extensive proliferation of mobilome-related elements (e.g., insertion sequences, integrase, and transposases) (Figure 4), but the locations of these mobile genetic elements do not fully explain the disruption of synteny (Figure 3).

Although CL-1 is most closely related to SCTE542, these two strains still show considerable divergence in genomes. The ANI value was only 91.2%, below the suggested cutoff of 95% for within-species comparison (Jain et al., 2018). Additionally, SCTE542 appeared to have undergone extensive genome degradation, such that it contains 944 annotated pseudogenes (Table 1) and lacks 641 genes shared by all other genomes compared (Figure 1D). Among the Japanese strains, *T. elongatus* BP-1 and *T. vulcanus* NIES-2134 are very similar in their genomes. These two strains share 96.5% of their genomic segments, and these segments have 99.0% ANI value (Figure 1C). Moreover, these two strains share ∼97% annotated genes (Figure 1D) and are unique compared with other *Thermosynechococcus* strains in their mobilome proliferation (Figure 4).

Taken together, these results suggest that some revisions of the *Thermosynechococcus* taxonomy may be necessary based on the 95% ANI cutoff (Jain et al., 2018). First, CL-1 likely represents a novel species within this genus. Second, SCTE542 is sufficiently divergent from BP-1 (i.e., ANI = 87.7%) to be considered as another novel species, rather than *T. elongatus*. Third, NIES-2134 has a highly similar genome and a close phylogenetic relationship to BP-1 (i.e., ANI = 99.0%) and thus could be re-classified as a strain of *T. elongatus*.

### Horizontal Gene Transfer

**FIGURE 5:**
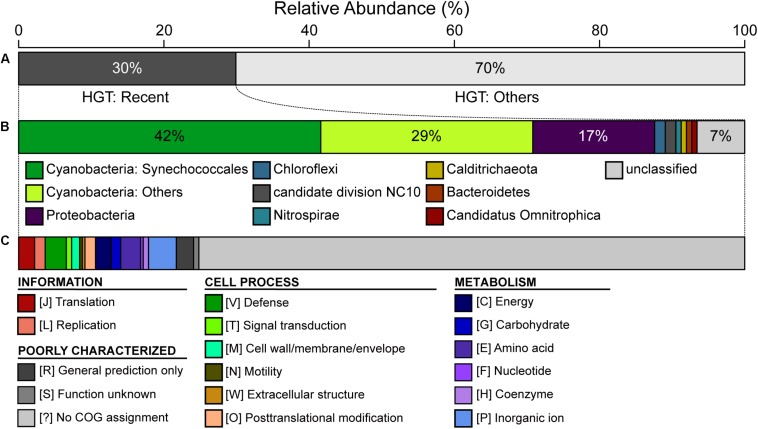
Summary of putative horizontal gene transfer (HGT). Among the 2,465 annotated protein-coding genes in the CL-1 genome, 458 were classified as putatively acquired by using a high-throughput BLASTP-based screening. **(A)** Classification based on the best hit; those with the best hit from outside of the genus *Thermosynechococcus* (137/458 = 30%) were classified as being recent acquisitions. **(B)** Taxonomic assignment of putative donors for recent HGT events. **(C)** Functional classification of recently acquired genes based on the COG database.

The CL-1 genome is notable in having a high number of putatively acquired genes. Based on the high-throughput BLASTP-based screening, 458 of the 2,465 (19%) annotated protein-coding genes may have been acquired (Supplementary Table S2). Among these, 137 have the best hit from a putative donor that does not belong to the genus *Thermosynechococcus*, which suggests that these are recent acquisitions. For these 137 recently acquired genes, 42% of the putative donors are from the order Synechococcales, 29% are from other more divergent Cyanobacteria, and 17% are from the phylum Proteobacteria (Figure 5). In terms of functions, 72% of these do not have specific category assignment based on COG. For those with functional assignment, inorganic ion transport and metabolism (i.e., category “P”) is the most abundant category, followed by amino acid transport and metabolism (i.e., category “E”) (Figure 5).

**FIGURE 6:**
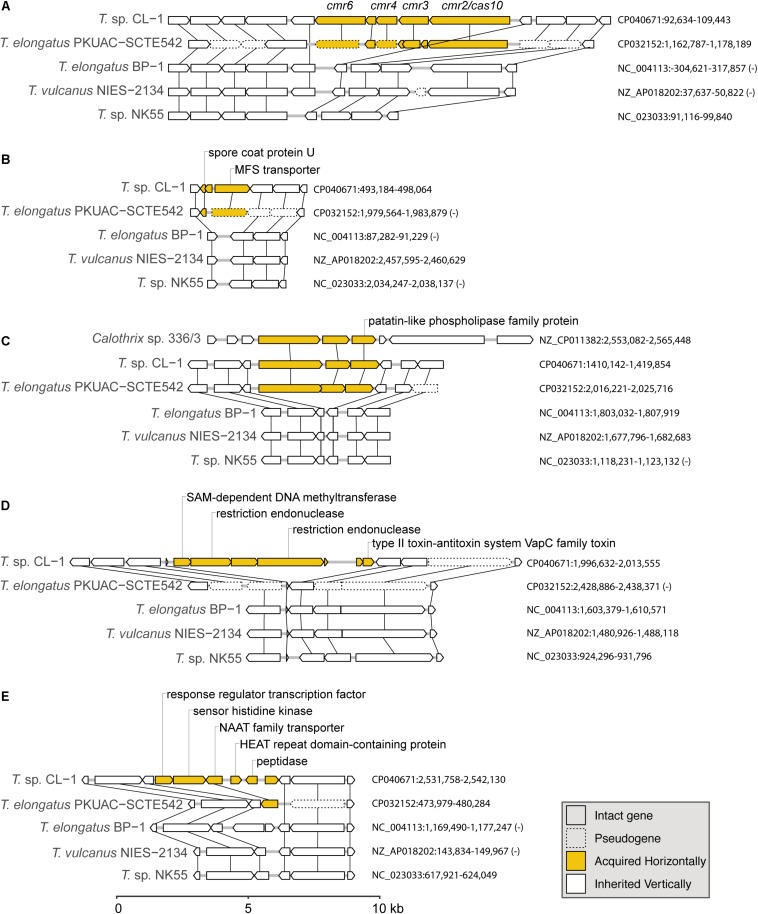
Examples of gene islands acquired by the CL-1 strain through horizontal gene transfer: **(A)** type III-B CRISPR-Cas system; **(B)** spore coat protein U and MFS transporter; **(C)** patatin-like phospholipase family protein; **(D)** multiple restriction endonucleases and type II toxin–antitoxin system; **(E)** NAAT family transporter. The sequence accession numbers and exact locations of these regions are labeled on the right. Genes in these regions are drawn to scale, and pseudogenes are drawn with dashed lines. Homologous genes across different genomes are linked by vertical lines. Putative acquired genes are in yellow. Locus tags of putatively acquired genes in the CL-1 genome: **(A)** FFX45_00470-FFX45_00490, **(B)** FFX45_02450-FFX45_02460, **(C)** FFX45_07120-FFX45_07130, **(D)** FFX45_09855-FFX45_09885, and **(E)** FFX45_12430-FFX45_12455.

Because this high-throughput BLASTP-based approach may not be reliable due to biases in the taxon sampling of available genomes in the current database, we manually inspected these results to examine the hits and synteny information. Notable examples are visualized (Figure 6 and Supplementary Figure S1) and discussed in more detail below.

The first example of gene acquisition is the type III-B CRISPR-Cas system found in CL-1 and SCTE542 genomes (Figure 6A; Makarova et al., 2011). The type I system in CL-1 and SCTE542 may have been inherited vertically because this system is also found in NK55. In contrast, the type III-B system may have been acquired horizontally in the common ancestor of CL-1 and SCTE542 (Figure 6A). Although the absence of these genes in the other three *Thermosynechococcus* genomes may be explained by one loss event, BLAST searches using the CL-1 homolog revealed that the best hits were from other families (i.e., not Synechococcaceae, which contains the genera *Thermosynechococcus*/*Synechococcus*) in the order Synechococcales. On the basis of this pattern, the absence of these genes in the most recent common ancestor (MRCA) of *Thermosynechococcus*, followed by horizontal gene acquisition in the MRCA of CL-1 and SCTE542 appears to be a reasonable hypothesis. However, this putatively acquired type III-B system lacks the CRISPR repeats and several other genes typically associated with the system (e.g., *cmr1* and *cmr5*). Thus, this partial type III-B system probably is not functional.

Two other examples of gene acquisition at the same phylogenetic branch involve one gene island containing a spore coat protein U and a major facilitator superfamily (MFS) transporter (Figure 6B) and another one containing a patatin-like phospholipase family protein (Figure 6C). In the latter case, the same gene island was found in *Calothrix* sp. 336/3, a hydrogen-producing cyanobacterium isolated from a lake in Finland (Isojärvi et al., 2015). However, the *Calothrix* phospholipase homolog has a low level of sequence similarity (i.e., 48% identity and 64% similarity in protein sequences), which indicates that either *Calothrix* is a distant relative of the putative donor, or the gene acquisition in CL-1 occurred in the distant past.

Furthermore, we found one region of putative acquisition specific to CL-1. This region contains multiple restriction endonucleases and a set of type II toxin-antitoxin systems (i.e., *vapBC*) (Figure 6D). Although *vapC* was also found in SCTE542 (locus tag: D3A95_07350) and NK55 (locus tag: NK55_RS09295), these genes are located in different regions of the chromosome and may not share the same evolutionary origin. Finally, an island with six putatively acquired genes was found in CL-1 (Figure 6E). One of these genes encoding for a NAAT family transporter was also present in SCTE542, so we do not know whether this region was acquired in the common ancestor of these two strains (followed by degradation in SCTE542) or whether CL-1 experienced multiple acquisition events in this region.

Finally, a glycosyltransferase gene in the CL-1 genome (Locus tag: FFX45_09045) may have been acquired from a Proteobacteria donor (Supplementary Figure S1). Although distant homologs of this gene could be found in other *Thermosynechococcus* genomes, those homologs form a strongly supported monophyletic clade that is quite distant from the CL-1 homolog. Instead, the CL-1 homolog is more closely related to those found in Proteobacteria (e.g., *Halomonas* and *Altererythrobacter*).

### Photosynthesis

**FIGURE 7:**
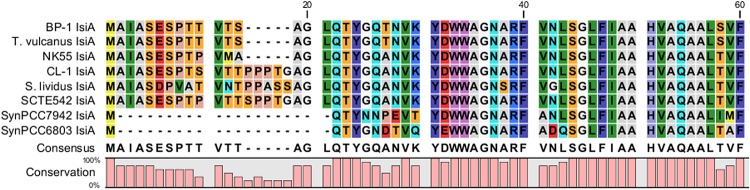
Comparative analysis of N-terminus amino acid sequences of iron stress-inducible (IsiA) proteins among five *Thermosynechococcus* strains, *S. lividus*, and two mesophilic cyanobacteria (i.e., *Synechococcus* sp. PCC7942 and *Synechocystis* sp. PCC6803) by using CLC Main Workbench (Qiagen Bioinformatics).

The major structural components of photosynthesis genes are well conserved among *Thermosynechococcus* genomes. For example, two copies of cytochrome c-550 genes (*psbV1*and *psbV2)* (FFX45_09625, FFX45_09620) were tandemly arranged in genomes of all *Thermosynechococcus* and *S. lividus* strains. In addition, three copies of *psbA* genes (*psbA1*, *psbA2*, and *psbA3*) (FFX45_08385, FFX45_08390, FFX45_10905) encode the reaction center D1 protein of photosystem II. *psbA1* and *psbA2* were tandemly arranged in genomes of all *Thermosynechococcus* strains except SCTE542. In the SCTE542 genome, *psbA1* and *psbA2* are separated by a transposase gene. Only two copies of *psbA* genes (*psbA1* and *psbA3 homologs*) were detected in the *S. lividus* genome. However, we found some significant variations in regulatory components of photosynthesis-related genes. For example, *sbtA*, the high-affinity sodium-dependent bicarbonate transport family permease gene, is present in only the genomes of CL-1 (FFX45_07280), SCTE542, and *S. lividus* but not in the three Japanese strains. In contrast, the high-affinity ABC-type bicarbonate transport system (encoded by *cmpABCD* operon) is present in the genomes of CL-1 (FFX45_03210-03225) and the three Japanese strains but not SCTE542 or *S. lividus.* Thus, only CL-1 has both types of high-affinity bicarbonate transporters and may have higher capacity for bicarbonate uptake under different growth environments. In addition, CL-1, SCTE542 and *S. lividus* have a distinct flavodoxin gene (*fldA*) (FFX45_02905), which is adjacent to a Crp/Fnr family transcriptional regulator gene (FFX45_02900) and may form an operon together. Flavodoxins are electron transfer proteins that may substitute the function of ferredoxin in the photosynthetic electron transport chain under iron-deficient conditions. Furthermore, iron stress-inducible proteins (IsiAs) are giant chlorophyll–protein complexes induced by iron deficiency in cyanobacteria (Bibby et al., 2001; Boekema et al., 2001). We found longer predicted N-terminal amino acid sequences of IsiA gene products in the three Japanese strains (with 15 extra amino acid residues) and CL-1, SCTE542 (pseudogene), and *S. lividus* (with 20 extra amino acid residues) than those of mesophilic cyanobacteria such as *Synechocystis* sp. PCC6803 and *Synechococcus* sp. PCC7942 (Figure 7). The structural differences among these IsiA proteins are consistent with the genome-scale phylogeny analysis that CL-1 from Taiwan is most closely related to SCTE542 from China, whereas the three other *Thermosynechococcus* strains from Japan form a sister clade. The physiological significance of structural differences among these IsiA proteins requires further study.

### Metabolism

The core metabolism genes are highly conserved among *Thermosynechococcus* genomes and are similar to those found in other cyanobacteria (Beck et al., 2012). In addition, all the *Thermosynechococcus* genomes lack nitrogenases and hydrogenases (Stolyar et al., 2014). Furthermore, all the *Thermosynechococcus* genomes have a complete gene set (*nrtABCD*) of the nitrate transport system, nitrate reductase, and nitrite reductase for the assimilation of nitrate. Of note, CL-1 appeared to lose a complete gene set (*urtABCDE*) of the urea ABC transport system and several important components of urease genes (*ureA*, *ureD*, and *ureG*), which are well conserved in the genomes of the other *Thermosynechococcus* strains. The CL-1 strain is likely unable to import and utilize urea as a nitrogen source. In addition, the CL-1 genome has a distinct gene operon for nitric oxide protection, which is absent in genomes of the other *Thermosynechococcus* strains. The gene cluster contains *dnrN* or *norA* [encoding an iron-sulfur cluster repair di-iron protein (FFX45_09820) putatively involved in the repair of nitrosative damage to iron-sulfur clusters] and *norB* [encoding nitric-oxide reductase large subunit (FFX45_09815)]. This gene operon may participate in nitrogen metabolism and in defense against nitric oxide toxicity.

### Transporters

CL-1, SCTE542, and *S. lividus* genomes have two distinct sets of gene clusters for the phosphate-specific transport-system *pstSCAB operon* (FFX45_00090-FFX45_00105, FFX45_04225-FFX45_04235). One shares high sequence similarity with the other *Thermosynechococcus* strains, but the other has a different origin. The multiple transporters for phosphate may have distinct affinity and/or activity under different phosphate concentrations. In addition, the CL-1 genome has two different types of Mg^2+^ transporter genes: *mgtE* (FFX45_01105) and *corA* (FFX45_03065). The SCTE542 genome has *mgtE*, and NK55 has *corA*. Moreover, CL-1, SCTE542, and *S. lividus* genomes have one gene operon that encodes a copper-translocating P-type ATPase (CopA) (FFX45_10400) and a Cu^+^ chaperone (CopZ) (FFX45_10405). The water analysis showed a small amount of arsenic (∼0.0183 mg/L) and nickel ions (0.212 mg/L) in the hot-spring water (see Supplementary Table S3). CL-1 and *S. lividus* genomes have one copy of an ArsB/NhaD family transporter (*arsB*) (FFX45_09985), but the other strains do not. Moreover, CL-1, NK55, and *S. lividus* genomes have one distinct gene operon that encodes the organoarsenical efflux MFS transporter ArsJ (FFX45_03530) and an associated glyceraldehyde-3-phosphate dehydrogenase (FFX45_03525). The ArsJ gene in the CL-1 genome exhibited 58% amino acid sequence identity to the ArsJ gene from *Pseudomonas aeruginosa* that conferred arsenate As(V) resistance (Chen et al., 2016). In addition, the CL-1 genome has one distinct MFS transporter gene (FFX45_02460; Figure 6B) that exhibited a significant degree of similarity to the nickel resistance gene (*nreB*) from *Synechocystis* sp. PCC6803 (54% amino acid sequence identity). Overall, CL-1 appeared to have several distinct transporter genes and various heavy metal efflux systems to cope with different stress conditions in hot-spring environments.

### Signal Transduction

For the two-component signal transduction system, 15 and 26 potential genes for His kinases and response regulators, respectively, were identified in the CL-1 genome. Most are conserved in all *Thermosynechococcus* genomes. For example, the CL-1 genome contains two sets of motility-related (PixJ and PilJ homolog-associated) two-component signaling systems [including signal transduction His kinase/response regulators CheA (FFX45_00335 and FFX45_12785), methyl-accepting chemotaxis proteins (FFX45_00340 and 12790), CheW proteins (FFX45_00345 and FFX45_12795), and response regulator (CheY) proteins (FFX45_00350, FFX45_12800, and FFX45_12805)] that are conserved among all *Thermosynechococcus* strains except *T. vulcanus*. PixJ homolog-associated (blue-light-responsive) two-component signaling systems are truncated in the *T. vulcanus* genome. Of note, NK55 and *S. lividus* genomes have two additional sets of chemosensory two-component signaling systems, and BP1 and SCTE542 have one additional set. In addition, GGDEF/EAL domain proteins function as diguanylate cyclases/phosphodiesterases that synthesize/degrade cyclic di-GMP and participate in a cyclic-di-GMP signaling pathway that may regulate biofilm formation, motility, virulence, and cell cycle (Agostoni et al., 2013). Recent studies identified several cyanobacterichromes with GGDEF/EAL domains that mediated blue-light-induced cell aggregation in BP1 and *T. vulcanus* (Enomoto et al., 2015). The CL-1 and *S. lividus* genomes have 13 GGDEF/EAL domain protein genes, and the other *Thermosynechococcus* genomes only have 9–10 genes (Table 2). The hot-spring cyanobacteria JA-3-3-Ab and JA-2-3Ba (in the A/B lineage) have only four GGDEF or EAL domain proteins (see Table 2 and Agostoni et al., 2013). Thus, *Thermosynechococcus* and *S. lividus* strains (in the C1 linage) seem to have more complex cyclic-di-GMP signaling pathways than JA-3-3-Ab and JA-2-3Ba (in the A/B linage).

**TABLE 2:** Number of GGDEF and EAL domain-containing proteins in five *Thermosynechococcus* strains and representative *Synechococcus* strains from Yellowstone hot springs.

Strain	GGDEF	EAL	GGDEF + EAL	Total
*T*. sp. CL-1	6	1	6	13
*T. elongatus* PKUAC-SCTE542	4	0	6	10
*T. elongatus* BP1	4	0	5	9
*T. vulcanus* NIES-2134	5	0	5	10
*T*. sp. NK55	5	0	5	10
*S. lividus* PCC 6715	5	0	8	13
*S*. sp. JA-3-3Ab	3	1	0	4
*S*. sp. JA-2-3Ba	4	0	0	4

### Chaperone–Usher System

CL-1, SCTE542, and *S. lividus* genomes have a unique gene cluster for a chaperone/usher system that is absent in the three Japanese strains. This gene cluster contains a molecular chaperone (*fimC*) (FFX45_06910), a fimbrial biogenesis outer membrane usher protein (*fimD*) (*FFX45_06905*), and three spore coat protein U domain-containing proteins (FFX45_06900, FFX45_06915, and FFX45_06920) (Geibel and Waksman, 2014). The chaperone/usher system is mainly present in proteobacteria but is also found in a few cyanobacteria. The function of the chaperone/usher system is to assemble proteinaceous filaments on the cell surface. These filaments could form fimbrial (pili) or non-fimbrial surface structures (e.g., a capsule or spore coat) (Leng et al., 2011).

### Bipartite Aminoglycoside Nucleotidyltransferases

The CL-1 and SCTE542 genomes have three and one copies of putative bipartite aminoglycoside nucleotidyltransferase gene operons (FFX45_05120, FFX45_05125, FFX45_07775, FFX45_07780, FFX45_11395, and FFX45_11400 for CL-1; AXY68115.1 and AXY68116.1 for SCTE542) that may confer kanamycin resistance (Lehmann et al., 2003). Most kanamycin nucleotidyltransferases (KNTases) are a homodimer with each subunit composed of two domains (one for substrate biding, the other for nucleotide binding). However, the bipartite aminoglycoside nucleotidyltransferase gene operons of CL-1 and SCTE542 contain two different genes (one encodes a substrate binding domain and the other nucleotide binding domains of KNTases). CL-1 can grow in medium containing kanamycin (5 μg/mL). In contrast, BP-1 does not have the KNTase gene and was susceptible to kanamycin.

### Prokaryotic Immunity Systems

The CL-1 and SCTE542 genomes encode both type I (FFX45_00470-00490) and type III-B CRISPR-Cas systems (FFX45_02320-02350) that confer resistance to foreign genetic elements (Makarova et al., 2011). The type I CRISPR-Cas system is also found in NK55. In addition, the CL-1 genome has type III restriction modification system (FFX45_09855, FFX45_09860, FFX45_09870, and FFX45_08355) in defense against foreign DNA molecules. BP1 and *T. vulcanus* have type I and type III restriction modification systems; the SCTE542 genome has type II restriction modification systems; and NK55 and *S. lividus* genomes both have type I to type III restriction modification systems. Furthermore, Cl-1 and NK55 genomes contain a distinct *vapBC* operon (FFX45_09880 and FFX45_09885) of the type II toxin–antitoxin system (Figure 6D). VapC is a toxin that induces RNA cleavage and is inhibited by the co-expression of the antitoxin VapB.

### Other Distinct Features in the CL-1 Genome

One distinct feature in the CL-1 genome is the presence of 14 copies of RPN family genes that encode recombination-promoting nuclease/putative transposases (Kingston et al., 2017). Most are located in two tandem gene arrays (nine and four copies, respectively) (FFX45_10335-10375 and FFX45_07895-07910) except for one gene (FFX45_07205). However, there are only eight, seven, three and two copies of Rpn family gene(s) in *T. vulcanus*, BP1, SCTE542, and NK55 genomes, respectively. In addition, RPN family genes are absent in genomes of *S. lividus*, JA-3-3-Ab and JA-2-3Ba from springs of Yellowstone National Park. Furthermore, CL-1 and SCTE542 genomes share several distinct genes for a PQQ-dependent sugar dehydrogenase (FFX45_00065), a linear amide C-N hydrolase (FFX45_04715), an ADP-ribosylglycohydrolase family protein (FFX45_09525) and a gene cluster for pseudaminic acid biosynthesis (FFX45_00685-00695, FFX45_00730, and FFX45_00735). The physiological significance of these distinct genes in the CL-1 genome may require further study.

## Conclusion

This work reported the comparative genomic analysis of a novel thermophilic cyanobacterium, *Thermosynechococcus* sp. CL-1, together with four other *Thermosynechococcus* strains and the outgroup *S. lividus* in the C1 genotype. Although the sequences of the 16S rRNA gene among these *Thermosynechococcus* strains are highly similar, the genome structures of these *Thermosynechococcus* strains exhibit extensive rearrangements. The genome-scale phylogenetic analysis and genome-wide ANI results both suggest that CL-1 is most closely related to SCTE542, and both are sufficiently divergent from other *Thermosynechococcus* lineages to be considered new species within this genus. In addition, we identified distinct genetic differences between CL-1 and the other *Thermosynechococcus* strains. Our results suggest that *Thermosynechococcus* strains actively acquired many functional genes via horizontal transfer to cope with the various types of stresses in alkaline hot springs.

## Data Availability Statement

The genome sequence reported in this work was deposited in GenBank (accession no. CP040671).

## Author Contributions

H-TH provided the biological materials. Y-IC, LC, and Y-FC performed the experiments. Y-IC, LC, Y-FC, C-HK, H-TH, and H-AC analyzed the data. Y-IC, LC, H-TH, C-HK, and H-AC wrote the manuscript. C-HK and H-AC acquired the funding and supervised the project.

## Conflict of Interest

The authors declare that the research was conducted in the absence of any commercial or financial relationships that could be construed as a potential conflict of interest.

## Footnotes

1 https://www.epa.gov.tw/niea-en/

## Supplementary Material

FIGURE S1 Maximum-likelihood phylogeny of a putatively acquired glycosyltransferase gene (Locus tag: FFX45_09045). Bootstrap values above 70% are labeled. The CL-1 homolog is distantly related to other *Thermosynechococcus* homologs, while more closely related to homologs from Proteobacteria (e.g., *Halomonas* and *Altererythrobacter*).

TABLE S1 Homologous gene clusters among representative thermophilic cyanobacteria genomes. Each row in the table lists one protein-coding gene; the cluster id (as assigned by OrthoMCL), and other information (e.g., genomic location and annotation) are included. Genes sharing the same cluster id are considered as homologs. Genes without any identifiable homolog were assigned to their own clusters with unique ids (i.e., singletons).

TABLE S2 Top five BLASTP hits in the NCBI non-redundant protein database of all annotated protein-coding genes in the CL-1 genome. The putatively acquired genes are highlighted (red: recent; blue: other).

TABLE S3 Chemical analysis of the water sample in the Chin-Lun hot spring.
