# Supplementary figures and images for: Comparative Genomic Analysis of a Novel Strain of Taiwan Hot-Spring Cyanobacterium Thermosynechococcus sp. CL-1
Source: Front Microbiol. 2020 Jan 31;11:82. doi: 10.3389/fmicb.2020.00082 (PMC7005997; doi:10.3389/fmicb.2020.00082)

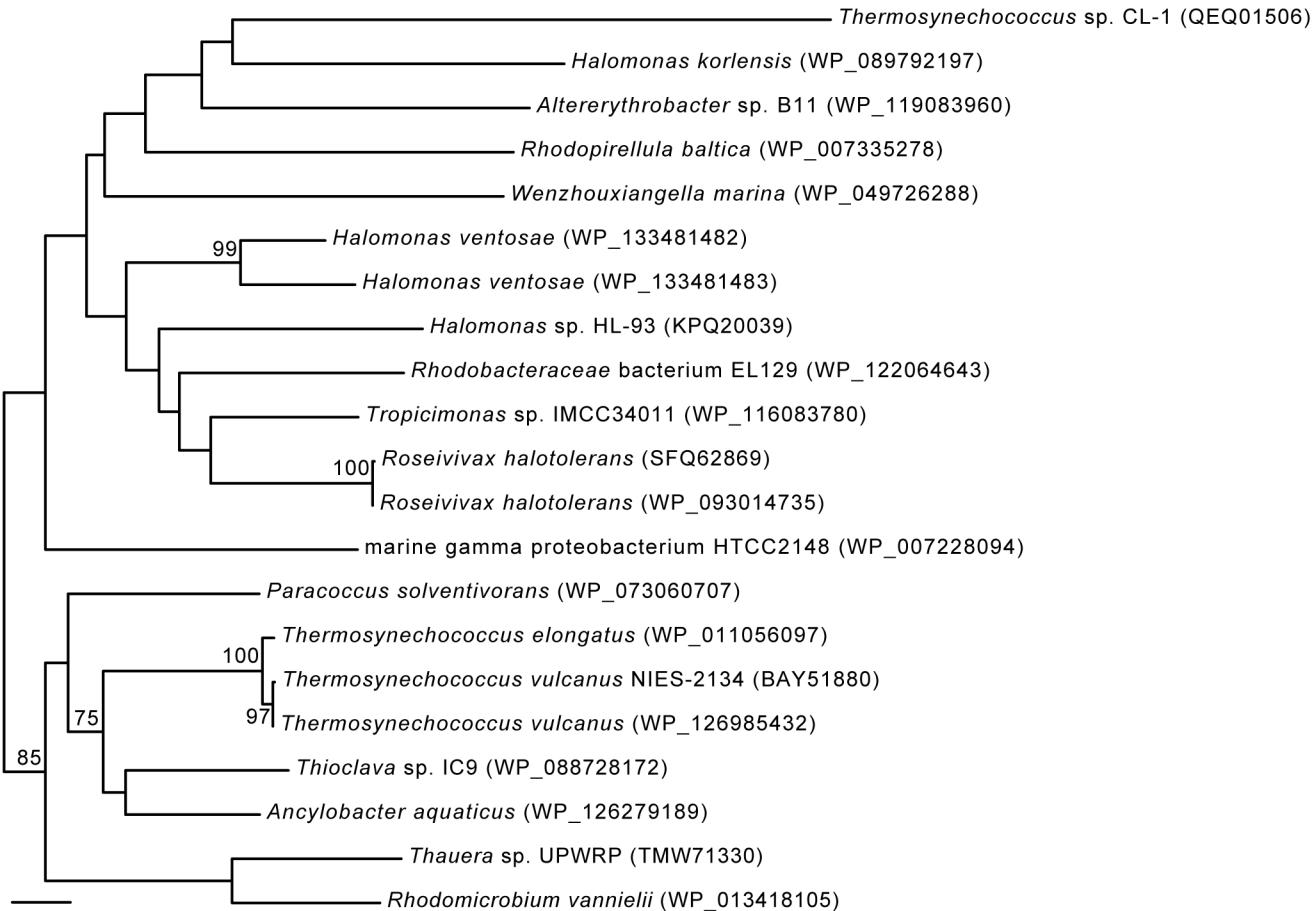

Supplement: FIGURE S1 — Maximum-likelihood phylogeny of a putatively acquired glycosyltransferase gene (Locus tag: FFX45_09045). Bootstrap values above 70% are labeled. The CL-1 homolog is distantly related to other Thermosynechococcus homologs, while more closely related to homologs from Proteobacteria (e.g., Halomonas and Altererythrobacter). [file Image_1.PDF]
