# Supplementary material for: Comparative Genomic Analysis of a Novel Strain of Taiwan Hot-Spring Cyanobacterium Thermosynechococcus sp. CL-1
Source: Front Microbiol. 2020 Jan 31;11:82. doi: 10.3389/fmicb.2020.00082 (PMC7005997; doi:10.3389/fmicb.2020.00082)
Supplement: TABLE S3 — Chemical analysis of the water sample in the Chin-Lun hot spring. [file Table_3.PDF]

Supplementary Table S3. Chemical analysis of the water sample in the Chin-Lun hot spring.

Item	Conc.	Note
Heavy metals	Unit (mg/L)	
As	0.0183	
Hg	<0.0005	MDL = 0.00021
Cr	ND	MDL = 0.0016
Ni	0.212	
Cu	ND	MDL = 0.0032
Zn	<0.010	MDL = 0.0030
Major ions	Unit (mg/L)	
Na^+	98.0	
NH_4^+	5.40	
K^+	7.00	
Mg^{2+}	1.55	
Ca^{2+}	8.64	
Cl^-	14.7	
NO_3^-	0.30	
PO_4^{3-}	26.6	
HCO_3^-/CO_3^-	18.8	
SO_4^{2-}	64.2	
Others		
NPOC	26.9 mg/L	
pH	10.39@82.9^{O}C	Temperature was 82.9^{O}C with in situ measurement
Conductivity	1.96 mS/cm	

Note: ND means "not detectable"; MDL means "method detection limit"
